# Agitated saline test as a simple but reliable method of intraoperative diagnosis and evaluation of unroofed coronary sinus

**DOI:** 10.1186/s40981-024-00709-0

**Published:** 2024-04-25

**Authors:** Tomohiro Yamamoto, Teppei Yamada, Yutaka Seino, Kyo Hayama, Shuichi Shiraishi

**Affiliations:** 1 Division of Anesthesiology, Niigata University Graduate School of Medical and Dental Sciences, Niigata, 951-8510 Japan; 2 Division of Thoracic and Cardiovascular Surgery, Niigata University Graduate School of Medical and Dental Sciences, Niigata, Japan

To the Editor,

Unroofed coronary sinus (URCS) is a general term for diseases with septal defects between the coronary sinus and left atrium [1]. The URCS often merges with the persistent left superior vena cava (PLSVC) [2]. The left atrium (LA) and right atrium (RA) are trafficked by the URCS, and the pressure gradient between them results in a left–right shunt at the atrial level via the URCS. Therefore, patients with URCS have hemodynamics similar to patients with atrial septal defects (ASD). While it is well known, its reported frequency is very low [3, 4]; therefore, not only anesthesiologists but also cardiac surgeons have few opportunities to manage patients with URCS. URCS may be detected when the coronary sinus (CS) and right heart system are enlarged; however, the fistula is often difficult to detect using transthoracic echocardiography [5]. Transesophageal echocardiography (TEE), particularly three-dimensional TEE, is reportedly superior in the diagnosis of URCS [6].

Here, we report the management of intraoperative anesthesia in a 16-year-old male patient with partially URCS without subjective symptoms, which was suspected due to a heart murmur and incomplete right bundle branch block on electrocardiography. Preoperative TEE noted an enlarged CS with two suspected defects of 15.4 mm × 9.2 mm and 25.8 mm × 9.9 mm opening to the LA, as well as right heart system loading findings. Based on these findings, surgery was performed. Preoperative computed tomography revealed a defect in the innominate vein and PLSVC flowing into the CS. These findings indicated that this case was a type 3 URCS with PLSVC or even possibly type 1 [2].

After the induction of general anesthesia, TEE was performed, which revealed an enlarged CS and structures suggestive of a URCS fistula; however, color Doppler imaging failed to reveal any obvious shunt flow (Fig. 1a, b). When 20 mL of agitated saline was vigorously administered from an intravenous line to the left upper extremity, it was easily observed that the agitated saline flowed from the CS into the LA (Fig. 1c, d). The cardiac surgeon reached the LA through right atrial and atrial septal incisions, and the fistula was closed directly (Fig. 2). An agitated saline test from the left upper extremity was repeated after URCS repair, which easily and reliably confirmed the absence of residual leakage from the CS into the left ventricular system (Fig. 3). The agitated saline test does not require any special procedures, such as mixing air; it only involves vigorous intravenous administration of the solution.

**Fig. 1:**
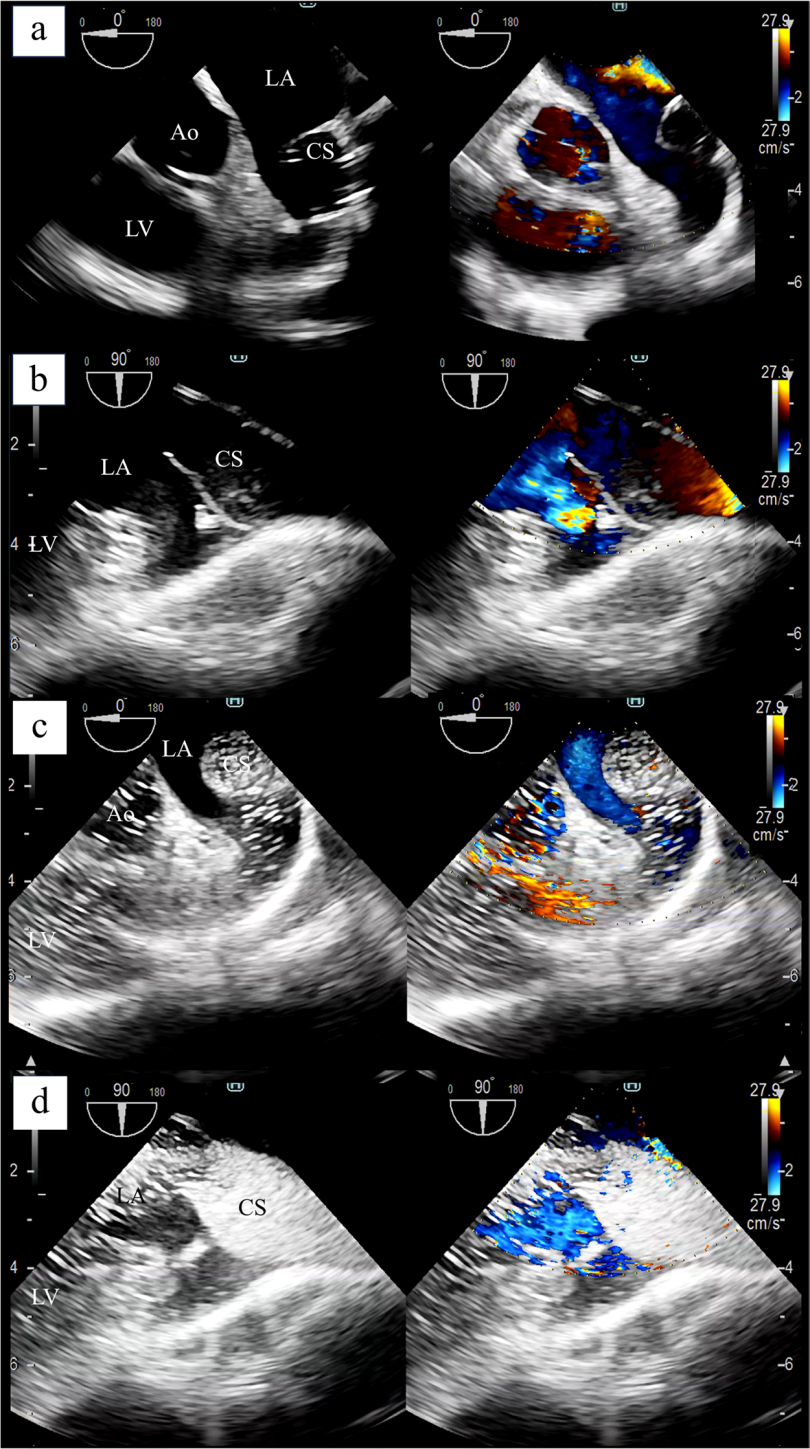
Intraoperative transesophageal echocardiography and agitated saline test before unroofed coronary sinus repair. Intraoperative transesophageal echocardiography (TEE) revealed an enlarged coronary sinus (CS) and structures suggestive of an unroofed coronary sinus (URCS) fistula in the **a** short-axis and **b** long-axis views. However, color Doppler imaging failed to detect any obvious shunt flow. When 20 mL of agitated saline is vigorously administered via an intravenous line from the left upper extremity, the agitated saline flowing from the CS into the left atrium can be easily observed (**c** and **d**). CS, coronary sinus; LA, left atrium; LV, left ventricle; Ao, ascending aorta

**Fig. 2:**
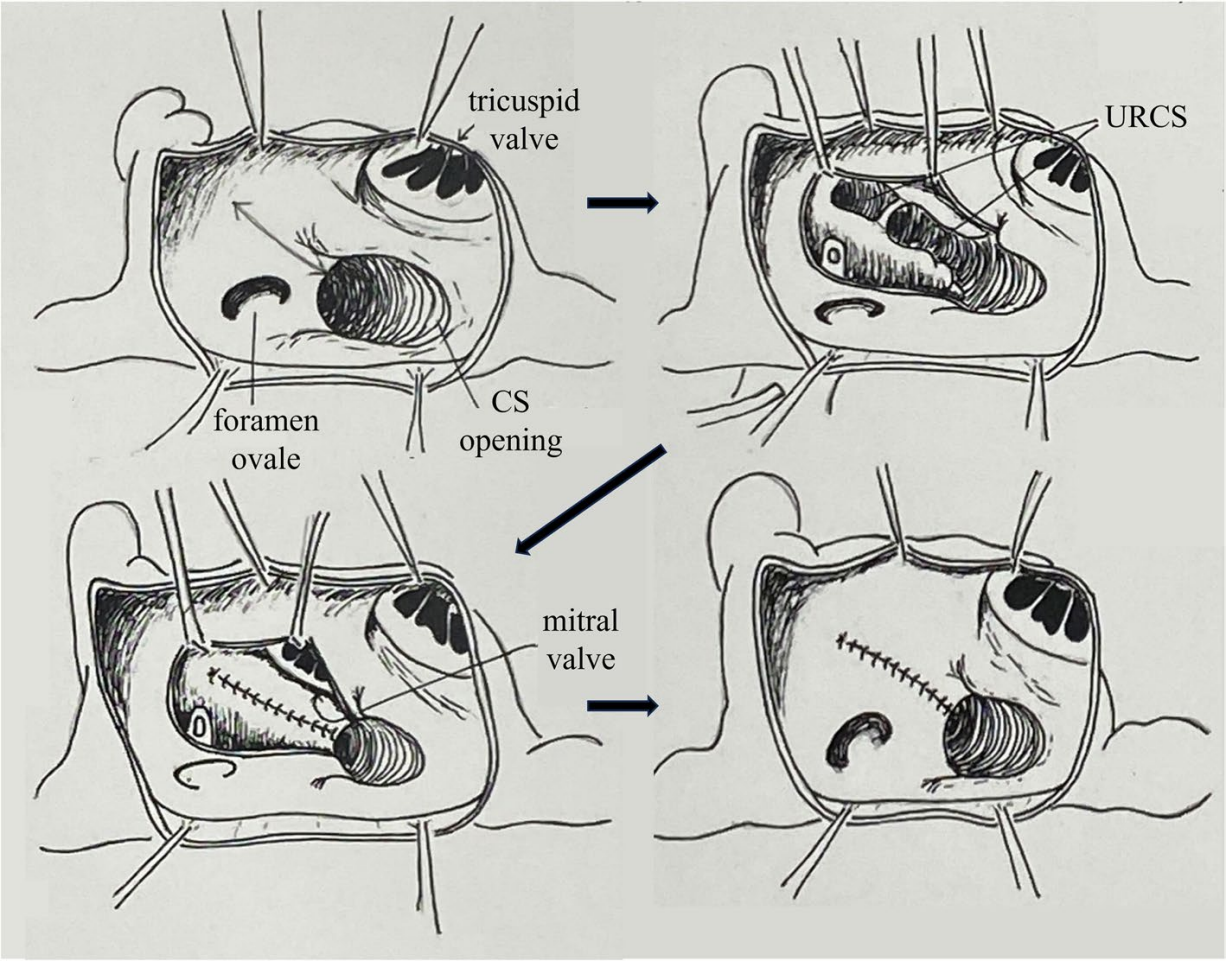
Schema showing surgical procedure of direct closure of unroofed coronary sinus fistula. The cardiac surgeon reached the left atrium through right atrial and atrial septal incisions, and the fistula was closed directly. CS, coronary sinus; URCS, unroofed coronary sinus

**Fig. 3:**
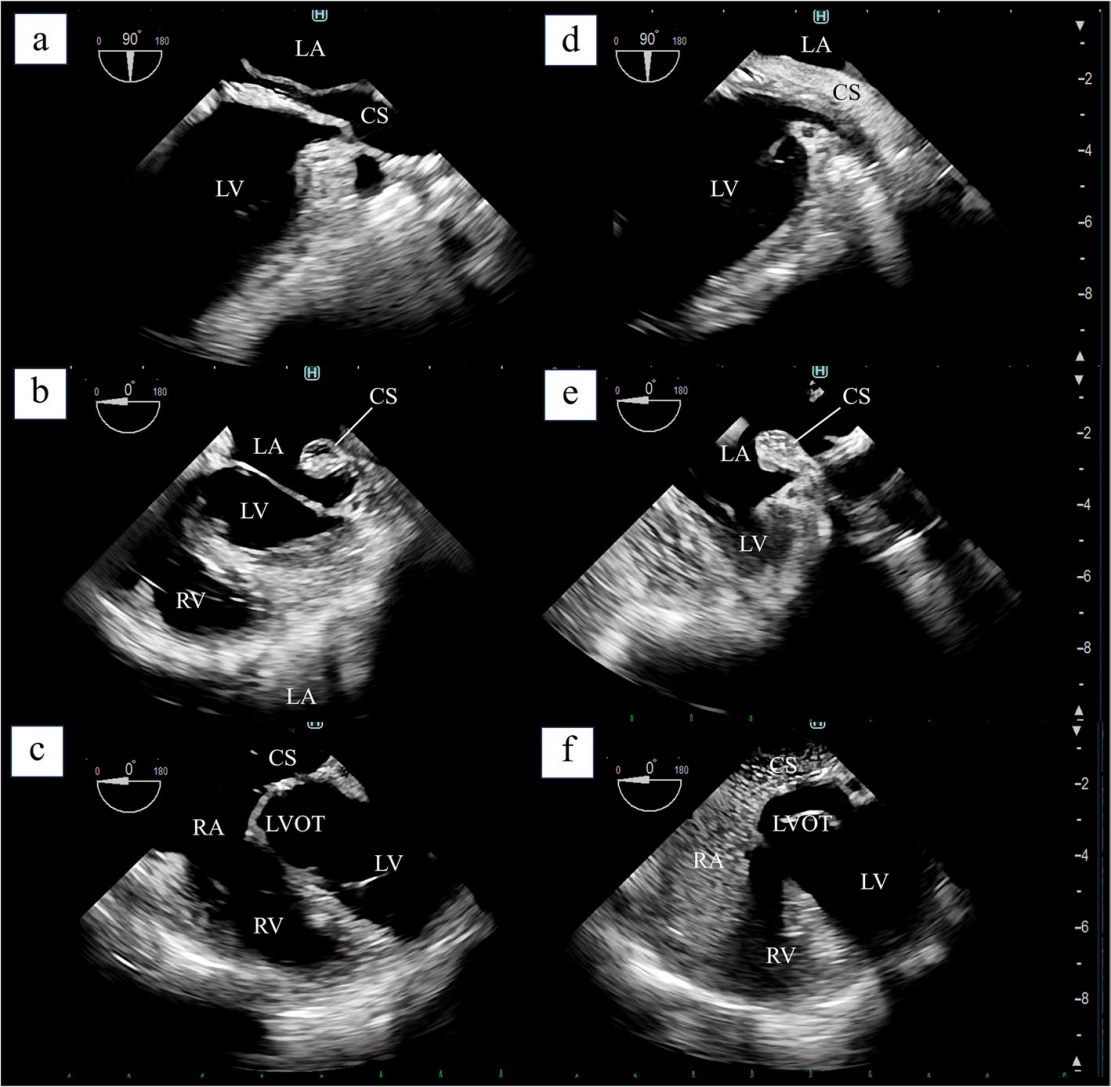
Intraoperative transesophageal echocardiography and agitated saline test after unroofed coronary sinus repair. **a** Short-axis view and **b** long-axis view of URCS after direct fistula closure. **c** Close to the five-chamber view with an enlarged CS opening to the right atrium. **d**, **e**, **f** The agitated saline test was performed again after URCS repair from the left upper extremity, which easily and reliably confirmed that there was no residual leakage from the CS into the left ventricular system. CS, coronary sinus; LA, left atrium; LV, left ventricle; RA, right atrium; RV, right ventricle; LVOT, left ventricular outflow tract

In URCS, the left–right shunt flow is slow owing to the small pressure gradient between the LA and RA. In addition, this pressure gradient becomes even smaller due to positive pressure ventilation during general anesthesia, and it becomes more difficult to detect the shunt flow using color Doppler imaging. We present a simple but reliable method to diagnose URCS and evaluate the efficacy of URCS repair by performing an agitated saline test from the left upper body during intraoperative anesthesia management for URCS complicated by PLSVC.

## Abbreviations

URCS: Unroofed coronary sinus
PLSVC: Persistent left superior vena cava
LA: Left atrium
RA: Right atrium
ASD: Atrial septal defect
CS: Coronary sinus
TEE: Transesophageal echocardiography
